# Polymer Additives with Gas Barrier and Anti‐Aging Properties Made from Asphaltenes via Supercritical Ethanol

**DOI:** 10.1002/advs.202307619

**Published:** 2023-12-13

**Authors:** Zulin Wu, Xiangbo Liu, Chao Ma, Mingjin Du, Xiangdong Ding, Changsheng Xiang

**Affiliations:** ^1^ State Key Laboratory for Mechanical Behavior of Materials Xi'an Jiaotong University 28 West Xianning Road Xi'an Shaanxi 710049 China

**Keywords:** asphaltene, composite, gas barrier, anti‐aging

## Abstract

Asphaltene is often regarded as an undesirable by‐product of petroleum processing, possesses vast reserves with little market value. The typical routes of consuming asphaltene, namely burning and landfilling, pose significant environmental challenges. In this study, low‐value asphaltene is converted into high‐value ethylated carbon clusters (ECC) using a supercritical ethanol technique. The resulting ECC powder demonstrates promising properties for high density polyethylene (HDPE) composite applications. The effects of incorporating ECC on the mechanical, gas barrier, and anti‐aging properties of the composite are investigated. Results show that a 1 wt.% ECC led to a 4.2% and 43.5% increase in tensile strength and elongation at break, a reduction of 45.8% and 30.7% in oxygen and carbon dioxide permeability. Furthermore, ECC exhibits effective UV spectrum absorption and conversion in the wavelength range of 400–600 nm, providing protection against UV spectrum damage to HDPE. The incorporation of ECC not only enhances the properties of polymer composites but also sequesters carbon within the polymer matrix, enabling the valorization of asphaltene while mitigating environmental impact.

## Graphical abstract

Explore the untapped potential of asphaltene, an undesired byproduct of petroleum processing. Conventional disposal methods (burning, landfilling) pose environmental challenges. Discover how supercritical ethanol technology offers a scalable solution, transforming asphaltenes into innovative polymer additives with enhanced gas barrier and anti‐aging properties, thereby enabling the valorization of asphaltene and mitigating environmental impact.

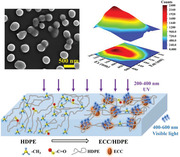

## 1 Introduction

Asphaltene is a complex chemical compound and a significant component of crude oil.^[^ ^1^ ^]^ Extensive research has shown that asphaltene can impede the production and transportation of crude oil, making its separation from crude oil a critical step for the petroleum industry.^[^ ^2^, ^3^ ^]^ The separation methods include solvent extraction,^[^ ^4^ ^]^ membrane filtration,^[^ ^5^, ^6^ ^]^ and supercritical fluid extraction.^[^ ^7^ ^]^ Asphaltenes are commonly used in the form of asphalt or bitumen products as paving materials on roads and waterproof coatings. Due to the vast daily oil production around the world, this undesired by‐product is often regarded as a waste and being disposed through tailing pond injection, leading to environmental concerns.^[^ ^8^ ^]^ Consequently, one of the key issues that needs to be resolved is how to improve the utility of asphaltene.

The increasing demand for hydrocarbon materials and resources has prompted a shift in the perception of asphaltene from a low‐value by‐product to a valuable raw material. Its low cost, natural abundance, and unique chemical characteristics make it an attractive option for high‐value applications. Researchers and industries are actively exploring ways to maximize the utility of asphaltene and unlock its potential in advanced applications. One area where asphaltene is gaining attention is in the synthesis of nanomaterials. Researchers have discovered that incorporating asphaltene as a filler in polymers can significantly improve the electrical, thermal, and mechanical properties of polymer composites.^[^ ^9^, ^10^, ^11^ ^]^ For instance, Mohammad^[^ ^12^ ^]^ enhanced the thermal characteristics of polystyrene (PS) by adding asphaltene. With only 2 wt.% asphaltene, PS's glass transition and degradation temperatures rose from 66 °C and 432 °C to 69 °C and 447 °C, respectively. At the same time, asphaltene can also be produced into various carbonaceous materials through different methods, such as activated carbon,^[^ ^13^ ^]^ carbon microspheres,^[^ ^14^ ^]^ fibers,^[^ ^15^ ^]^ and graphene.^[^ ^16^, ^17^ ^]^ For example, Saadi successfully converted asphaltene into flash graphene via the flash joule heating process. The asphaltene‐derived flash graphene showed improved mechanical, thermal, and anti‐corrosion properties for the composites compared to the bare polymer.^[^ ^17^ ^]^

Research on the structure of asphaltene has revealed that it primarily consists of polycyclic aromatic hydrocarbons with alkane branches, as well as other heteroatoms such as O and N.^[^ ^8^, ^18^ ^]^ In this study, we employed the supercritical method to assemble nano‐scale asphaltene units into ethylated carbon clusters (ECC). This assembly was achieved through molecular electrostatic attraction and π–π^*^ interaction. The alkane branched chain structure of ECC facilitates its uniform dispersion in polyolefin materials. ECC exhibits a layered structure resembling graphene. In previous studies, Karolina et al.^[^ ^19^ ^]^ prepared composites of graphene nanosheets (1 wt.%)/low density polyethylene using the melting blending method, resulting in a 34.7% decrease in CO_2_ permeability. Similarly, Honaker et al.^[^ ^20^ ^]^ prepared composites of graphene nanosheets (15 wt.%) without surface modification using the melting blending method, leading to a 73% reduction in O_2_ permeability in high density polyethylene (HDPE) composites. These findings suggest that ECC may effectively reduce gas permeability. Furthermore, ECC possesses the ability to absorb UV light and convert it into visible light within the range of 400–600 nm. These clusters offer advantages such as reduced gas permeability and enhanced mechanical and anti‐aging performances. They hold promise for utilization in various industries, including oil and gas pipelines and packaging.

## 2 Results and Discussion

### 2.1 Structure, Composition, and Morphology of ECC

**Figure 1:**
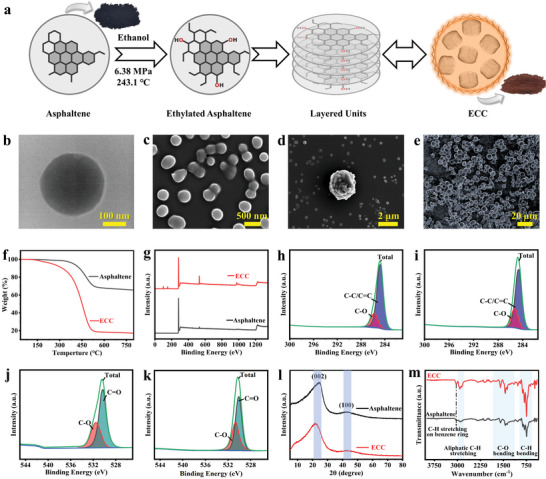
The synthesis procedure and characterizations of ECC. a) Schematic illustration of the synthesis of ECC (red powder) from asphaltene (black powder). b) TEM images of ECC at 10 ppm. c,d) SEM images of ECC dispersion in ethanol at a concentration of 500 ppm. e) SEM image of ECC powder. f) TGA of asphaltene and ECC conducted in the N_2_ atmosphere with a ramp rate of 10 °C min^−1^. g) XPS survey spectral comparison of asphaltene and ECC. XPS high‐resolution C1s spectra of h) asphaltene and i) ECC. XPS high‐resolution O1s spectra of j) asphaltene and k) ECC. l) XRD spectra of asphaltene and ECC. m) FT‐IR spectra of asphaltene and ECC.

In addition to having a high potential for refining heavy crude oil through both catalytic and non‐catalytic processes, supercritical alcohols have a high enough chemical reactivity to change heavy organic compounds and add various heteroatomic functional groups to their molecular structures.^[^ ^21^ ^]^ Ethanol that is over its critical point (6.38 MPa, 243.1 °C) is referred to as supercritical ethanol. The hydrogen bonds of ethanol get weaken as it reaches a supercritical state, which makes it a potent solvent for gases, organic materials, and other substances.^[^ ^22^ ^]^ A scheme is created to better comprehend how asphaltene transforms into ECC (**Figure** **1**a). In the supercritical state of ethanol, asphaltenes are functionalized into ethylated asphaltenes, with ethyl groups on the edges. Under the influence of Van der Waals force, these ethylated asphaltene molecules accumulate into layered units. These layered units are further coalesced together and rolled into spherical clusters by the action of surface tension in order to maintain the system's stability and reach the lowest energy state.^[^ ^23^ ^]^ After treating asphaltene with supercritical ethanol, the color of the powder change from black to red, as shown in Figure 1a. The proposed mechanism was further proved by the microscopic characterization. Transmission electron microscope (TEM) image shows the asphaltene accumulate into circular layered units (Figure 1b). Their average size is calculated to be 130 nm (Figure S1a,b, Supporting Information). The height of the layered units is characterized with atomic force microscope (AFM) (Figure S1c, Supporting Information), and the average thickness was determined to be 16.7 nm (Figure S1d, Supporting Information). The TEM and AFM measurements confirmed that the asphaltenes accumulate into a disk‐like structure. As the reaction continues under the supercritical ethanol condition, the asphaltene layered units coalesce with each other as shown in the scanning electron microscope (SEM) analysis (Figure 1c) and rolled into spherical clusters by surface tension, forming spherical ECC (Figure 1d). Upon further treatment under the supercritical condition, more spherical clusters are formed at the micrometer scale as shown in Figure 1e, and the average size of ECC is calculated to be 5.15 µm (Figure S2, Supporting Information). It is worth to mention that the ECC can be reversed back into the ethylated asphaltene units when they are re‐dispersed in ethanol at a low concentration. In order to characterize the size of ECC in ethanol at various dispersion concentrations, particle sizes of ECC at different concentrations (10, 50, 100, 250 ppm) was measured (Figure S3a, Supporting Information). The size of ECC decreases as the dispersion concentration gets lower, which suggests ECC can reverse back into ethylated asphaltene units. To elucidate the cause of this phenomenon, electrode potential experiments on the ECC dispersed in ethanol was performed. The results show that ECC contained both positive and negative charges (Figure S3b, Supporting Information). The average electrode potential was 7.8 mV, which suggested that ECC was formed by electrostatic forces between asphaltene units. At higher concentration, the asphaltene units tends to accumulate and rolled into ECC. However, when the concentration of asphaltene units get lower, the electrostatic force is not strong enough to bind the asphaltene units so they tend to pull apart from each other; few ECC was formed under this scenario. According to the SEM pictures (Figure S4, Supporting Information), there are not only nanoscale spheres (Figure S4a, Supporting Information ) but also micrometer‐scale structures (Figure S4b, Supporting Information) in the ECC precipitation. However, Figure S4b (Supporting Information) shows that there are no nanoscale spheres around the micrometer‐scale structure, and this micrometer‐scale structure is clearly composed of nanoscale spheres, this is because the nanospheres are “absorbed” by the micrometer‐scale structure under the action of electrostatic forces, indicating that ECCs will attract each other and undergo self‐assembly phenomenon.

Thermogravimetric analysis (TGA) demonstrates that there are some key distinctions between asphaltene and ECC (Figure 1f). At 350 °C, asphaltene starts to decompose, and it undergoes a complete weight loss of 30% by 550 °C. In contrast, ECC starts to degrade ≈150 °C, and experienced a weight loss of 80% at 550 °C. This suggests that ECC are less stable than asphaltene and the cause may arise from the ethyl and hydroxyl groups on the edges of the ECC. X‐ray photoelectron spectroscopy (XPS) was used to determine the composition of ECC (Figure 1g). The oxygen percentage grew from 2.3% to 10.4% during the conversion process, mostly because oxygen atoms were introduced during the supercritical reaction. In addition, both asphaltene and ECC have no other heteroatoms. In order to better understand their chemical structure, high‐resolution XPS spectra of asphaltene and ECC were analyzed. The high‐resolution C1s spectrum of asphaltene can be fitted to two components centered ≈284.7 and 285.7 eV, namely C─C/C═C and C─O (Figure 1h); The high‐resolution O1s spectrum of asphaltene can be fitted to two components centered ≈530.3 and 531.5 eV, namely C═O and C─O (Figure 1j). On the other hand, the high‐resolution C1s peak of ECC can generate two peaks corresponding to C─C/C═C and C─O at 284.6 and 285.3 eV, respectively (Figure 1i); The high‐resolution O1s spectrum of ECC can be fitted to two components centered ≈530.1 and 530.6 eV, namely C═O and C─O (Figure 1k).

X‐ray diffraction (XRD) patterns of asphaltene and ECC showed a graphite‐like ordered structure (002) and a hexagonal ordered structure (100), respectively, both display two separate broad peaks (≈24° and 44°) (Figure 1l).^[^ ^24^ ^]^ Fourier‐transform infrared (FT‐IR) spectroscopy was performed to further analyze the composition changes between asphaltene and ECC, that they tend to share the same peak positions of the infrared spectra (Figure 1m). In the wavelength range of 600–900 cm^−1^, the C─H bending vibration was represented, whereas the characteristic peak of C─O bending vibration is represented by the wavelength range of 1100–1800 cm^−1^, the stretching vibration of aliphatic C─H, such as ─CH_3_ and ─CH_2_─, appears in the range of 2800–3000 cm^−1^, and the stretching vibration of the C─H on the benzene ring peaks at 3030 cm^−1^. Compared with asphaltene, the infrared absorption peak intensity of the C─O and C─H in ECC is higher, this is due to the reaction between asphaltene and ethanol in the supercritical state of ethanol, where hydroxyl and ethyl groups are introduced into the asphaltene, resulting in an increase in the content of C─O and C─H in ECC, which is consistent with the increase in oxygen percentage in XPS analysis.

### 2.2 Mechanical Properties of HDPE/ECC Composite

HDPE is a widely used polymer for pipelines or containers because of its advantages of light weight, corrosion resistance, and robust mechanical properties.^[^ ^25^ ^]^ HDPE/ECC composite was made via melt blending. Tensile strength and strain, storage modulus, loss modulus of the composites were tested at 0, 0.5, 1, and 3 wt.% ECC loadings.

**Figure 2:**
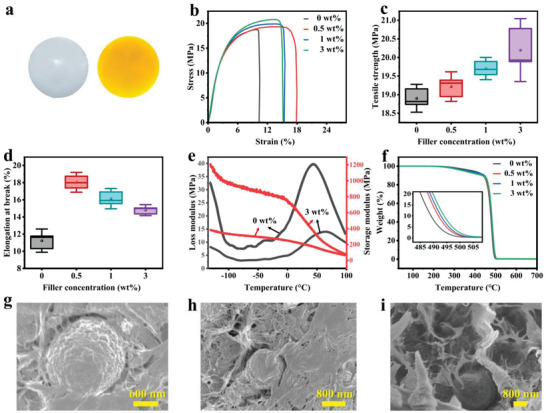
Mechanical and thermal properties of ECC/HDPE composites (prepared by melt‐blending). a) Photos of HDPE film (left) and 3 wt.% ECC/HDPE film (right). b) Stress–strain curves of ECC/HDPE composite films with different ECC loadings. Comparison of c) tensile strength and d) elongation at break of ECC/HDPE composite films with different ECC loadings (n ≥ 3). The lines inside the box represent the median, the circles represent the mean, the top and bottom lines represent the upper and lower limits of the 90% confidence interval, and the distribution interval of all the data is represented by the box. e) Temperature dependence of storage modulus and loss modulus of ECC/HDPE composite films with different ECC loadings. f) TGA results of ECC/HDPE composite film with different ECC loadings. SEM image of the cross‐section of 3 wt.% ECC/HDPE film processed by g) razor blade cutting; h) liquid nitrogen quenching; i) tensile fracture.

The ECC/HDPE composite film exhibits a yellow color, while the pure HDPE film appears semi‐transparent and white (**Figure** **2**a). Due to the existence of ethyl groups on the ECC edges, ECC can be uniformly dispersed in the HDPE matrix. Analysis of the stress‐strain curves of ECC/HDPE composite films with varying filling ratios reveals an increase in the tensile strength with higher ECC content (Figure 2b,c). Specifically, at a filling ratio of 3 wt.%, the tensile strength experiences a 6.9% enhancement compared to pure HDPE films. This improvement can be attributed to the strengthening of the interface bonding between ECC and HDPE. Regarding the elongation at break, it initially increases and then decreases as the ECC filling ratio rises (Figure 2d). This behavior can be explained by the fact that during the melt‐blending process at lower ECC loadings, only a small portion of HDPE molecular chains bind to ECC, allowing a greater mobility of the majority of HDPE chains. This increased mobility reduces the restriction between HDPE molecular chains, leading to a higher elongation at break. However, as the filling ratio of ECC continues to rise, the mobility of HDPE chains becomes limited, resulting in a greater binding of ECC with HDPE chains and subsequently causing a decline in elongation at break.

Figure 2e presents the storage modulus, loss modulus of the ECC/HDPE composite film. When ECC is incorporated into the HDPE film, the storage modulus increases while the loss modulus decreases in comparison to pure HDPE film. This indicates that the ECC/HDPE composite film exhibits higher stiffness and improved structural stability under dynamic loads. The enhancement in storage modulus is attributed to the effective transmission of stress between interfaces facilitated by the inclusion of ECC. As the filling ratio of ECC increases, the efficiency of stress transfer between interfaces improves, leading to increased storage modulus and stiffness of the ECC/HDPE composite film. The loss modulus of the ECC/HDPE composite film displays two mechanical relaxation processes as the temperature increases. The range of −130–100 °C corresponds to the γ relaxation process, which is associated with the movement of short branch chains on the main chain. The range of 0–100 °C corresponds to the α relaxation process, which is related to the orientation and folding of molecular chains in the PE crystal region and lamellar slip mechanism, this α relaxation process partly reflects the material's glass transition temperature.^[^ ^26^ ^]^ Compared to pure HDPE, the ECC/HDPE composite film prepared through the melting method exhibits increased height and amplitude of the γ relaxation peak, indicating enhanced binding of molecular segment motion and an increased number of molecules undergoing transformation in the amorphous region. In the temperature range of 0–100 °C, the ECC/HDPE composite film shows a significant α mechanical relaxation process, with the peak shifting toward higher temperatures, indicating that ECC enhances the crystallinity of HDPE.

The TGA thermogram of the ECC/HDPE composite film (Figure 2f) reveals that the addition of ECC has little effect on the initial thermal decomposition temperature of HDPE, all the ECC/HDPE composite film and pure HDPE film start to break down ≈300 °C. The breakdown occurs at a faster rate as the temperature increases. At 490 °C, the decomposition mass of pure HDPE film reached 91.8%, while the decomposition masses of the composite films with 0.5, 1, and 3 wt.% ECC added were 85.8%, 83.3%, and 80.5%, respectively. Compared to pure HDPE film, the decomposition mass of ECC/HDPE composite films decreased by 6.5% to 12.3%, indicating an improvement in the thermal stability of the composite film.

SEM images of the 3 wt.% ECC/HDPE composite film reveal the presence of micron‐sized, spherical ECC particles encapsulated within the composite and bonded the HDPE matrix (Figure 2g). The composite film was also quenched in liquid nitrogen and subsequently broke apart for SEM analysis. Figure 2h displays the cut sections of the fragmented composite film, clearly revealing the solid interior of the ECC. The cross‐section of composite film after tensile fracture was also observed under SEM, micron‐sized ECC are visible in the HDPE matrix (Figure 2i).

### 2.3 Gas Barrier Performance

Ensuring effective gas barrier performance is crucial for HDPE, particularly when it is utilized as a liner for oil and gas pipelines. The permeation of O_2_ and CO_2_ through the HDPE liner can lead to corrosion of the underlying steel pipes. Similarly, when HDPE is used for food and beverage packaging in the form of bottles, the permeation of gases becomes undesirable, as it can compromise the freshness and quality of the packaged contents. Therefore, enhancing the gas barrier properties of HDPE is of paramount importance in these applications. Considerable research is currently focused on enhancing the gas barrier properties of polymers by incorporating layered silicate, graphene and its derivatives, and other 2D materials as fillers.^[^ ^27^, ^28^ ^]^

**Figure 3:**
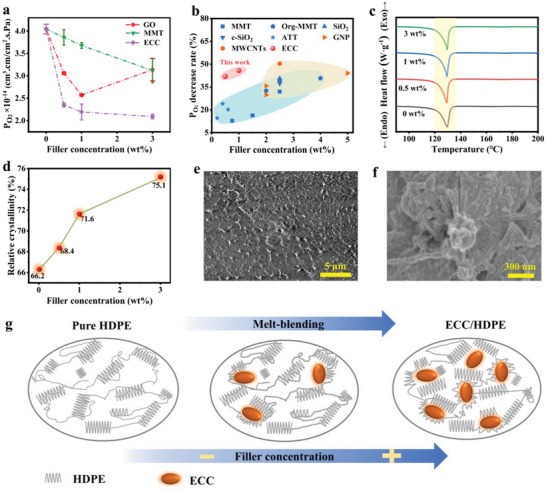
Characterization of gas barrier properties of composite films. Gas permeability of composite films with different filling ratios, a) O_2_. Measurement data are presented as mean ± standard deviation (SD) with n = 3. b) The comparison on gas barrier performance of ECC and other fillers reported in recent literature. c) DSC curves of ECC/HDPE composite film. d) Crystallinity of ECC/HDPE composite film. e) SEM image of 3 wt.% ECC/HDPE composite film. f) Zoom in SEM image of (e). g) Schematic illustration of the crystallinity of ECC/HDPE composite. ECC/HDPE (prepared by melt‐blending), GO/HDPE (prepared by solution‐blending), MMT/HDPE (prepared by ball‐milling).

This article investigates the impact of different fillers and filling ratios on the permeability of O_2_ and CO_2_ in HDPE films. The fillers used include layered silicate with montmorillonite (MMT), graphene oxide (GO) and ECC, which are composited with HDPE to produce a film (**Figure** **3**a; Figure S5, Supporting Information). In the case of GO/HDPE composite films prepared using the solution approach, the O_2_ and CO_2_ permeability initially decreases and then increases as the filling ratio increases. This behavior can be attributed to the tendency of GO to aggregate at higher filling ratios, leading to more defects in the HDPE films and increased O_2_ and CO_2_ permeability. However, even with the increased permeability, the GO filling still enhances the gas barrier performance of HDPE films compared to pure HDPE. For the MMT/HDPE composite film produced through the ball‐milling process, the O_2_ and CO_2_ permeability gradually decreases with an increase in filling ratio. At a filling ratio of 3 wt.%, the composite film exhibits a reduction of ≈22.3% in O_2_ permeability and 2.0% in CO_2_ permeability compared to HDPE film. This decrease can be attributed to the strong dispersibility of MMT and its gas‐blocking properties. In the case of ECC/HDPE composite films prepared using the melting process, the reduction in O_2_ and CO_2_ permeability is even more significant compared to MMT and GO fillers. The O_2_ permeability and CO_2_ permeability of ECC/HDPE composite films decrease by ≈45.8% and 30.7%, respectively, compared to HDPE films at a filling ratio of 1 wt.%.

This difference in permeability between O_2_ and CO_2_ can be explained by their respective dynamic diameters. The dynamic diameter of O_2_ (dO_2_ = 0.346 nm) is larger than that of CO_2_ (dCO_2_ = 0.330 nm). Additionally, oxygen requires more energy than carbon dioxide during the diffusion process, resulting in a lower diffusion coefficient for O_2_. According to the dissolution diffusion mechanism, the permeability coefficient is equal to the product of the dissolution coefficient and diffusion coefficient, leading to the observed lower O_2_ permeability compared to CO_2_ permeability in HDPE composite films.^[^ ^29^ ^]^

To further emphasize the excellent gas barrier properties of ECC in HDPE, we compared it with MMT,^[^ ^30^ ^]^ organically modified MMT (Org‐MMT),^[^ ^31^, ^32^ ^]^ SiO_2_,^[^ ^32^ ^]^ modified SiO_2_ (c‐SiO_2_),^[^ ^32^ ^]^ attapulgite (ATT),^[^ ^33^ ^]^ graphene nanoplatelets (GNP) ,^[^ ^20^, ^34^ ^]^ and multiwalled carbon nanotubes (MWCNTs).^[^ ^32^ ^]^ To make a fair comparison, all the HDPE composites were processed by melt mixing. As shown in Figure 3b, ECC maintains a significantly higher reduction in oxygen permeation even at low addition levels, surpassing clay materials (blue area). In contrast, carbon nanomaterials (orange area) achieve effects similar to ECC but require a higher addition level, resulting in much higher costs. It is evident that ECC offers substantial advantages on the efficiency of enhancing gas barrier performance of HDPE.

In comparison, we also examined the O_2_ and CO_2_ permeability of ECC/HDPE and asphaltene/HDPE composite films using the solution blending approach (Figure S6, Supporting Information). The results showed that at low filling ratios, asphaltene exhibited a reduction in both O_2_ and CO_2_ permeability. However, as the filling ratio increased, the O_2_ and CO_2_ permeability of asphaltene did not decrease but instead increased. This can be attributed to the tendency of asphaltene to aggregate and create more defects in the HDPE films, resulting in higher O_2_ and CO_2_ permeability. On the other hand, the O_2_ and CO_2_ permeability of ECC/HDPE composite films prepared using the solution approach consistently decreased with increasing ECC loadings. At a filling ratio of 1 wt.%, the O_2_ permeability decreased by ≈34.3% and the CO_2_ permeability decreased by ≈19.4%. Furthermore, it is noteworthy that ECC/HDPE composite films produced using the melt‐blending method exhibit superior gas barrier properties compared to films produced using the solution‐blending method and the ball‐milling method (Figure S7, Supporting Information). This is advantageous for the large‐scale practical applications of ECC/HDPE films since the melt‐blending method is not only cost‐effective but also environmentally friendly.

The ability of polymer films to act as gas barriers depends on their capacity to impede the passage of gas molecules. This capacity is influenced by factors such as the crystallinity of the polymer film, the size of the gas molecules, and the aspect ratio of the filler material. These factors collectively determine whether gas molecules can permeate through the thin film.^[^ ^35^ ^]^ To investigate the relative crystallinity of ECC/HDPE composite films produced via the melting process, the differential scanning calorimetry (DSC) curves of films with different filling ratios were analyzed (Figure 3c). The DSC curves provide insights into the crystalline structure and behavior of the films. By examining these curves, the relative crystallinity of the ECC/HDPE composite films at various filling ratios can be determined.

(1) $$\chi_{c}=\frac{\Delta H_{m}}{\Delta H_{m}^{0}}\;\;\times \;100\%$$

Where *H*_m_ is the melting point of the polymer, expressed in J g^−1^, and $H_{m}^{0}$ is the melting point of 100% crystallization of the polymer.^[^ ^36^ ^]^ Theoretically, polyethylene with100% crystallization has a melting enthalpy of 286.7 J g^−1[^ ^37^ ^]^ The calculations conducted in this study revealed a correlation between the filling ratio of ECC and the relative crystallinity of the ECC/HDPE composite film (Figure 3d). As the filling ratio of ECC increased, the relative crystallinity of the film also increased. For instance, when the filling ratio of ECC reached 3 wt.%, the relative crystallinity improved from 66.2% to 75.1% compared to pure HDPE film. This enhancement in relative crystallinity indicates that the incorporation of ECC raises the crystalline structure of the HDPE film, leading to a reduction in the permeability of O_2_ and CO_2_. This finding aligns with existing literature, which reports that increased polymer crystallinity can contribute to decreased gas permeability of materials.^[^ ^38^ ^]^ In the 3 wt.% ECC/HDPE composite film, ECC is uniformly dispersed within the HDPE matrix (Figure 3e), and upon closer inspection, ECC is observed to be wrapped by the HDPE chains (Figure 3f). The improved gas barrier performance of the composite film can be attributed to the ordered arrangement of HDPE molecular chains, initiated by the presence of ECC. This molecular arrangement is facilitated by the stretching of chain segments outward from the entangled molecular chains and the synergistic effects resulting from the addition of ECC during the film pressing process (Figure 3g). Overall, the incorporation of ECC into HDPE results in improved gas barrier performance due to the favorable molecular arrangement and interaction between the two materials.

### 2.4 Anti‐Aging Performance

Extended exposure to UV radiation can lead to the breakage of the molecular chains in HDPE, resulting in the formation of carbonyl and methyl groups.^[^ ^39^, ^40^ ^]^ These changes have adverse effects on the safety, durability, and mechanical properties of HDPE. This study also investigated the influence of ECC filling ratios on the aging resistance of HDPE.

**Figure 4:**
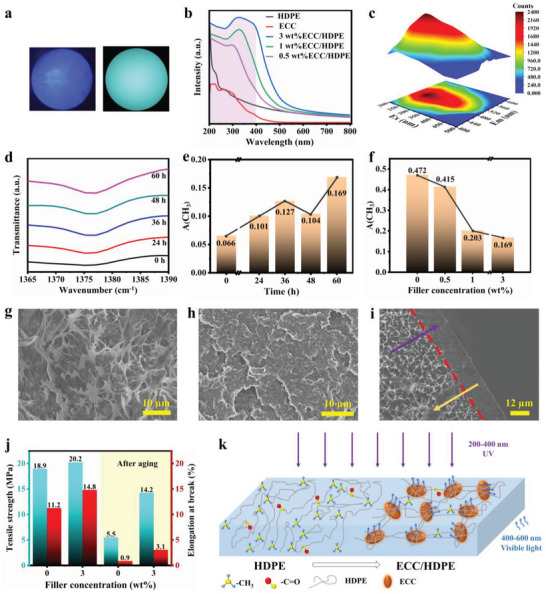
Anti‐aging performance of ECC/HDPE composites (prepared by melt‐blending). a) Photo of HDPE (left) and 3 wt.% ECC/HDPE (right) under 365 nm UV light. b) UV absorption spectra of HDPE, ECC and composite films. c) Excitation‐emission spectra of ECC and HDPE composite films. d) FT‐IR data of methyl groups in HDPE films upon UV exposure. e) Methyl group concentration of 3 wt.% ECC/HDPE film upon UV exposure for different time. f) Methyl group concentration of ECC/HDPE films after aging for 60 h. g) SEM image of HDPE h) SEM image of HDPE after aging for 60 h. i) SEM image of 3 wt.% ECC/HDPE after aging for 60 h (The red dashed line represents the boundary line, the purple arrow represents the direction of UV light irradiation, and the yellow arrow represents the direction of ECC movement). j) Mechanical properties of HDPE and its composite films before and after aging. k) Anti‐aging scheme of ECC/HDPE composite film.

Under UV irradiation, a noticeable blue–green fluorescence is observed in the 3 wt.% ECC/HDPE composite film, while the pure HDPE film does not exhibit any fluorescence (**Figure** **4**a). UV–vis spectrophotometer measurements were conducted to analyze the UV absorption properties of ECC and ECC/HDPE composite films. The results indicate that ECC possesses the ability to absorb UV light within the wavelength range of 200–400 nm, and this absorption capacity persists even after being compounded with HDPE (Figure 4b).

A red shift on the UV–vis absorption was observed when the ECC loading increase from 0 to 3 wt.% in the composite film. The dispersion of ECC in HDPE leads to changes in the interaction between ECC molecules and HDPE chains. The red shift becomes more prominent with an increasing ECC filling ratio. This can be attributed to the increased probability of self‐assembly as the ECC filling ratio rises, expanding the conjugated system of ECC molecules and influencing the conjugated structure of HDPE's unsaturated C─H bond on ECC.

Figure 4c shows the excitation and emission spectra of the 0.5 wt.% ECC/HDPE composite film, revealing its ability to absorb UV light while emitting visible light. By partially converting some of the UV light into visible light during the UV aging process, ECC helps mitigate the harmful effects of UV radiation on the HDPE substrate. Furthermore, the emission of the composite film is not dependent on excitation, consistently exhibiting a fluorescent emission peak at 510 nm regardless of the excitation wavelength. Overall, the results highlight the potential of ECC to enhance the UV resistance of HDPE films by absorbing UV light and converting it into visible light, thereby reducing the detrimental impact of UV radiation on the HDPE substrate.

Upon exposure to UV light, the microstructure of the ECC/HDPE composite film undergoes changes. In comparison to continuous network structure observed in the unaged microstructure of the pure HDPE film (Figure S8, Supporting Information), the pure HDPE film after UV aging treatment exhibits a discontinuous network structure (Figure S9, Supporting Information). This difference suggests that the molecular chains of HDPE are broken as a result of UV aging.

The aging process of HDPE under UV exposure primarily involves the breakage of its molecular chains and the formation of new groups through interactions with oxygen and water present in the environment. These newly formed groups include carbonyl and methyl groups. In this study, the aged ECC/HDPE composite film was examined using FT‐IR spectroscopy (Figure S10, Supporting Information). Since carbonyl groups were not significantly generated during aging, the methyl group was chosen as an indicator to assess the degree of aging in the composite film. To visually analyze the aging degree, the peak area corresponding to the methyl group was computed, reflecting the quantity of methyl groups present in the composite film. For pure HDPE, the peak area of the methyl group initially increased, then decreased, and subsequently increased again as the aging period progressed (Figure 4d). A similar trend in the peak area of the methyl group was observed when the filling ratio was 3 wt.% (Figure 4e). During the aging process, both fracture and cross‐linking of HDPE chains occur simultaneously,^[^ ^41^ ^]^ there is a competitive relationship between the effects of cross‐linking and fracture on the chemical composition and mechanical properties of HDPE.^[^ ^40^ ^]^

During the initial stages of aging, the presence of hydrogen ions in the environment results in the formation of methyl groups when the C═C bonds break. Consequently, the content of methyl groups increases with the duration of the aging process. As the content of methyl groups reaches a certain level, cross‐linking between methyl groups takes place under UV and high‐temperature conditions. This leads to the formation of shorter HDPE chains and a subsequent decrease in the content of methyl groups. Cross‐linking becomes predominant at this stage. As the content of methyl groups continues to decrease, chain breakage occurs to compensate for the reduction in methyl group content. To better assess the impact of ECC on the UV aging resistance of HDPE film, the change in methyl group content in the ECC/HDPE composite film with different filling ratios after aging for 60 h was analyzed (Figure 4f). The findings indicate that as the filling ratio increases, the content of methyl groups decreases, suggesting that the presence of ECC influences the aging resistance of HDPE film.

After the aging process, SEM was employed to characterize the morphology of ECC/HDPE composite films with different filling ratios. The unaged HDPE film exhibited a “ribbon‐like structure” from its polymer chains being pulled apart (Figure 4g). However, after aging, the pure HDPE film revealed a transition from a continuous “ribbon‐like structure” to “block‐like structure” (Figure 4h). This change can be attributed to the long molecular chains breakage into shorter chains that occurs during the aging of HDPE.

The microstructure analysis of the aged ECC/HDPE composite film reveals two distinct morphologies and structures. On the side of the film that is closer to the UV lamp, the HDPE molecular chains exhibit a characteristic “ribbon‐like structure.” In contrast, the side of the film that is away from the UV lamp displays a “block‐like structure” (Figure 4i). This difference in morphology can be attributed to the movement of ECC toward the UV lamp, as ECC prefers absorbing the UV spectrum. Under the influence of electrostatic forces, ECC undergoes displacement toward the UV lamp, leading to the observed differences in microstructure. Similar phenomena were also observed in the 1 wt.% ECC/HDPE composite film (Figure S11, Supporting Information). These findings provide further confirmation of ECC's ability to protect HDPE from aging effects caused by UV radiation.

To evaluate the resistance to aging, the mechanical characteristics of the pure HDPE film and the 3 wt.% ECC/HDPE composite film were compared before and after aging (Figure 4j). The addition of ECC improved the tensile strength of the HDPE composite film even without UV irradiation. At a filling ratio of 3 wt.%, the tensile strength of the HDPE film increased by 6.9%. Compared to the aged pure HDPE film, the 3 wt.% ECC/HDPE composite film showed a significant enhancement in tensile strength by 158.2%, and elongation at break was increased by 244.4% after 60 h of UV aging treatment. The tensile strength of the HDPE film decreased by 70.9%, while that of the 3 wt.% ECC/HDPE composite film decreased by 29.7%. The improved mechanical properties can be attributed to ECC's ability to absorb UV photons and convert them into visible light, thereby reducing the damage caused by UV radiation to HDPE. The effectiveness of UV absorption increases with an increasing ECC filling ratio, further enhancing the resistance to UV aging in the composite film.

The incorporation of ECC as a filler in HDPE mitigates the formation of methyl groups within the HDPE matrix, leading to a reduction in molecular chain breakage, as depicted in Figure 4k.

## 3 Conclusion

In conclusion, this study utilizes a supercritical approach to convert low‐value asphaltene into high‐value ECCs. These ECCs possess sheet‐like (nanoscale) and spherical (micrometer level) structures due to electrostatic attraction. By incorporating ECCs into the HDPE matrix through a melting process, they can be uniformly dispersed and wrapped within the HDPE molecular chains, resulting in a composite film with enhanced tensile strength and elongation at break. Furthermore, ECC's inherent gas barrier property and its synergistic effect on improving the relative crystallinity of HDPE materials contribute to the improved gas barrier performance of the HDPE films. Additionally, ECC exhibits the ability to absorb UV light and convert it into visible light, reducing the direct damage of UV radiation to the HDPE matrix and enhancing the film's anti‐aging properties. By significantly enhancing the safety and performance of polymers, ECC opens up new possibilities for various applications in oil and gas pipelines and packaging industries, as well as offering potential for the valorization of abundant reserves of asphaltenes.

## 4 Experimental Section

### Materials

HDPE (LH514, 0.956 g cm^−3^) was purchased from Taiwan Polymeric Chemicals Co. Ltd. (China). Asphaltene was obtained from Shandong Changren New Material Co. Ltd. (China). MMT (800 mesh) was purchased from the Lingshou Woao Mineral Products Processing Plant in Shijiazhuang (China). GO was synthesized using the improved Hummer's method.^[^ ^42^ ^]^ Xylene (AR, 99%) was acquired from Xi'an Haimeng Experimental Technology Co., Ltd. (China). Ethanol (AR, 99.7%) and acetone (HPLC, 99.7%) were purchased from China National Pharmaceutical Group Chemical Reagents Shaanxi Co., Ltd. (China).

### Preparation of ECC

Asphaltene (500 mg) and ethanol (40 mL) were added to the stainless steel reactor, and the temperature was raised to 285 °C to reach the supercritical state of ethanol (243.1 °C, 6.38 MPa). After 2 h of reaction, the reaction mixture was cooled to room temperature. Polytetrafluoroethylene with a pore size of 0.45 µm was used to filter it, and the filtrate was evaporated by rotation to obtain the ECC powder.

### Preparation of Asphaltene/HDPE, ECC/HDPE Composite Films

*Solution‐Blending*: To prepare the ECC/HDPE composite film, HDPE particles (4 g) and xylene (40 mL) were mixed in a three‐necked flask at 140 °C and 800 rpm for 1 h. In a separate beaker, ECC powder (20 mg) and xylene (10 mL) were stirred at room temperature and 800 rpm. The ECC/xylene mixture was then slowly added to the HDPE solution in the flask and stirred for 10 min at 800 rpm. The resulting solution was immediately transferred to a container with acetone (50 mL) and stirred for 10 min to allow settling. Rotary evaporation is employed to remove the organic solvent, resulting in a 0.5 wt.% ECC/HDPE composite powder. A small amount (1 g) of the composite powder was hot pressed at 150 °C and cut to obtain ECC/HDPE composite film discs with a diameter of 10 cm and a thickness of 100 ± 7 µm. ECC/HDPE composite films with filling ratios of 1 and 3 wt.% were prepared using the same procedure. The preparation of Asphaltene/HDPE composite thin film follows the same steps as mentioned above.

*Melt‐Blending*: To prepare the ECC/HDPE composite films, HDPE particles (40 g) were added to the sample chamber and melted using a torque rheometer at a temperature of 150 °C. Once the HDPE particles was completely melted, ECC (200 mg) powder was introduced into the chamber, and the temperature was reduced to 120 °C. The mixture is stirred at a speed of 20 rpm for 30 min before being removed to obtain the bulk 0.5 wt.% ECC/HDPE composite. A small portion (1 g) of the bulk composite material was then hot pressed at 150 °C and cut into film with a diameter of 10 cm and a thickness of 100 ± 7 µm. Following the same procedure, ECC/HDPE composite films with filling ratios of 1 and 3 wt.% were prepared. The preparation process for asphaltene/HDPE composite films follows these steps accordingly.

### Preparation of MMT/HDPE Composite Films

*Ball‐Milling*: To prepare the MMT/HDPE composite films, the HDPE particles were first crushed into powder using a crusher. Then, added the powdered HDPE (40 g) and MMT (200 mg) to a tank filled with crystalline balls. Ball mill the mixture at 300 rpm for 6 h. Removed the resulting powder to obtain a 0.5 wt.% MMT/HDPE composite powder. A small portion (1 g) of the composite powder was taken and was hot pressed at 150 °C. By adjusting the amount of MMT powder, MMT/HDPE composite films with filling ratios of 1 and 3 wt.% could be made using the same method.

### Preparation of GO/HDPE Composite Films

The procedure followed the same guidelines as preparing ECC/HDPE by the solution‐blending method.

### Accelerated Aging

During the film aging test, an UV aging chamber (ASRI, ACR‐2135) was utilized. The source of UV radiation is a Xenon‐arc lamp with 313 nm (91.5 kcal gmol^−1^) wavelength. The film must be aged, unless otherwise specified, at 50 °C and 0.5 W m^−2^·nm of radiation intensity.

### Characterization Methods

*Morphology Characterization*: Field emission scanning electron microscope (Hitachi, Hitachi SU8230) was used to characterize the asphaltene, ECC, and ECC/HDPE composite film and also to study the cross‐section of the ECC/HDPE composite film before and after UV aging. Pt was sputtered on all sample surfaces before imaging. Loren transmission electron microscope (Thermo) was used to characterize the ECC. The ECC was placed on a copper grid of 3 mm diameter and analyzed at an accelerating voltage of 200 kV. The surface morphology of ECC powder was observed by the tapping mode of atomic force microscope (Brooke, Dimension Edge). The particle size distribution of ECC was analyzed using ZETA potential and nanometer particle size analyzer (Marvin, Zeta sizer Nano ZSE).

*Structural Analysis*: FT‐IR spectroscopy (Bruker VERTEX70) was used to analyze the composition changes between asphaltene and ECC, as well as the composition changes of ECC/HDPE composite film before and after UV aging. The ordered structure of asphaltene and ECC was analyzed by using X‐ray diffractometer (Bruker D8 ADVANC) with Cu‐K_α_ radiation, and the 2*θ* values ranged from 5° to 90°. X‐ray photoelectron spectrometer (Thermo, EscaLab Xi+) was used to determine the elemental compositions of asphaltene and ECC. The XPS spectra in the 1350 to 0 eV binding energy range were recorded, and the energy step size was set to 1 eV with a pass energy of 100 eV.

*Thermal Analysis*: The thermal stability of the asphaltene, ECC, and ECC/HDPE composite film was analyzed with a synchronous thermal analyzer (TGA/DSC 3+). The testing was carried out from 25 to 800 °C at a heating rate of 10 K min^−1^ under a nitrogen atmosphere. DSC measurement was carried out on the differential scanning calorimeter (TA Instruments, DSC 250) from 25 to 200 °C at 10 °C min^−1^ under nitrogen.

Curves of damping factor, storage modulus, and loss modulus at different temperature were performed on dynamic thermodynamic analyzers (NETZSCH, DMA242M) of model DMA 242. The temperature rose from −130 to 100°C at a rate of 2 °C min^−1^, 1 Hz frequency, and 1% strain. The static force was set to 0.01 N, and the dynamic force was set to 0.1 N. The thin film sample was rectangular and had a cutting size of 30 mm × 6 mm × 0.1 mm.

*Mechanical Testing*: Stress and strain tests were performed using a dynamic mechanical analyzer (DMA) model DMA 850 by TA Instruments. The thin film sample had a cutting size of 30 mm × 6 mm × 0.1 mm, the strain rate was set to 1 mm min^−1^, and the preload force was set to 0.02 N.

*Gas Barrier Performance Testing*: The membrane gas (CO_2_, O_2_) permeability was performed with a differential pressure gas permeameter (Labthink Instruments Co., Ltd., VAC‐V2). The gas permeability must be measured at 23 °C and 0% relative humidity, unless otherwise specified. Everett digital thickness gauge is used to determine the film thickness. The film has a 10 cm diameter and a 100 ± 7 µm thickness. For each sample, the given effective gas permeability is the mean of three separate tests.

*Optical Performance Testing*: UV–vis spectrophotometer (Hitachi, UV‐1780) was used for spectral analysis of the HDPE, ECC, and composite films. The testing wavelength range is between 200 and 800 nm. Fluorescence spectrophotometer (Hitachi, F‐4700) was used for excitation‐emission spectra of ECC/HDPE composite films.

### Statistical Analysis

Error bars in all figures indicated the standard deviation over at least three tests, and each data were expressed as mean ± SD. All box plots illustrate the maximum value, minimum value, mean, median, and 90% confidence interval for all data points (n ≥ 3), with the specific representation method specified in the corresponding figure legend.

## Conflict of Interest

The authors declare no conflict of interest.

## Supporting information

Supporting Information

## Data Availability

The data that support the findings of this study are available from the corresponding author upon reasonable request.
