# Supplementary material for: Polymer Additives with Gas Barrier and Anti‐Aging Properties Made from Asphaltenes via Supercritical Ethanol
Source: Adv Sci (Weinh). 2023 Dec 13;11(9):2307619. doi: 10.1002/advs.202307619 (PMC10916603; doi:10.1002/advs.202307619)
Supplement: Supplementary file 1 — Supporting Information [file ADVS-11-2307619-s001.pdf]

ADVANCED SCIENCE
Open Access

Supporting Information

for *Adv. Sci.*, DOI 10.1002/advs.202307619

Polymer Additives with Gas Barrier and Anti-Aging Properties Made from Asphaltenes via Supercritical Ethanol

Zulin Wu, Xiangbo Liu, Chao Ma, Mingjin Du, Xiangdong Ding and Changsheng Xiang**

Supporting Information

Polymer Additives with Gas Barrier and Anti-Aging Properties Made from Asphaltenes via Supercritical Ethanol

Zulin Wu, Xiangbo Liu, Chao Ma, Mingjin Du, Xiangdong Ding*, Changsheng Xiang*

Z. Wu, X. Liu, C. Ma, M. Du, X. Ding, C. Xiang

State Key Laboratory for Mechanical Behavior of Materials

Xi'an Jiaotong University

28 West Xianning Road, Xi'an, Shaanxi, 710049, China

E-mail: dingxd@xjtu.edu.cn; c.xiang@xjtu.edu.cn

Microscopic morphology and size statistics of ECC

Silicon wafers were spin coated with an ethanol dispersion containing 100 ppm ECC, and the microstructure was examined using SEM. The size of ECC particles was calculated using ImageJ software, with a minimum count of 100 particles. Similarly, mica sheets were treated with an ethanol dispersion of 100 ppm ECC, and the thickness of ECC was measured using an AFM. To further investigate ECC properties, ethanol dispersions with concentrations of 10 ppm, 50 ppm, 100 ppm, 250 ppm, and 500 ppm ECC were prepared. The size of ECC particles at various concentrations and the potential of ethanol dispersions containing 250 ppm ECC were measured using a ZETA potential and nanoparticle size analyzer.

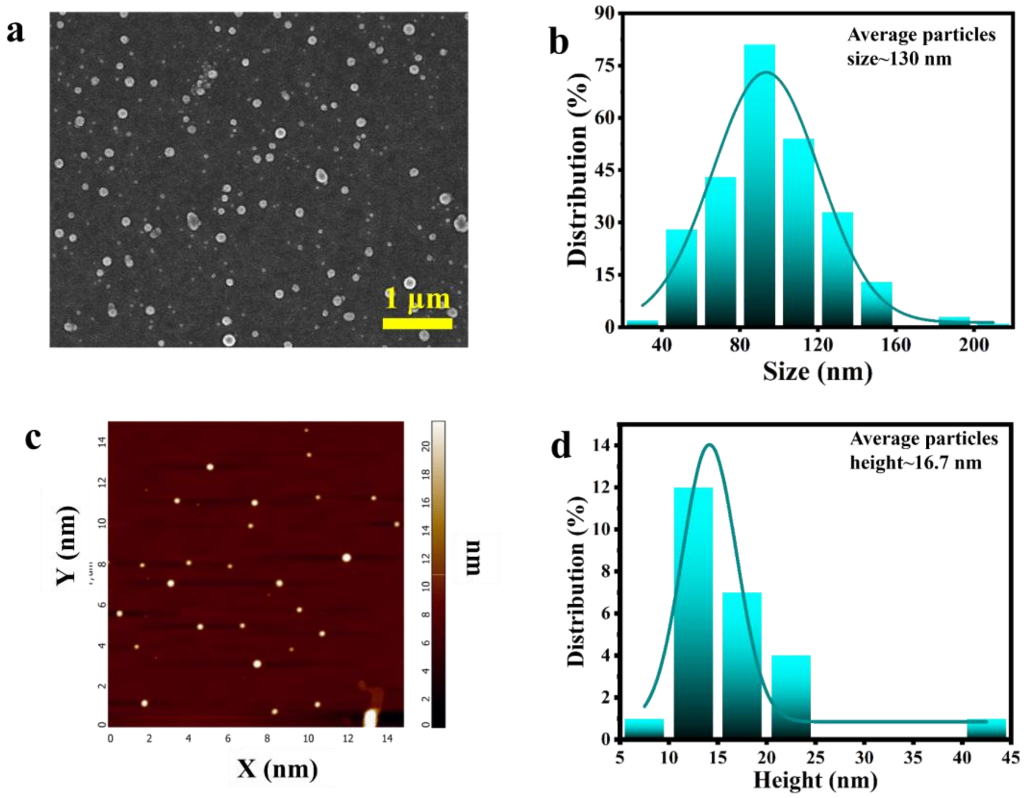

Figure S1. (a) SEM image of 100 ppm ECC in ethanol. (b) Particle size distribution of (a). (c) AFM image of 100 ppm ECC in ethanol. (d) Thickness distribution of (c).

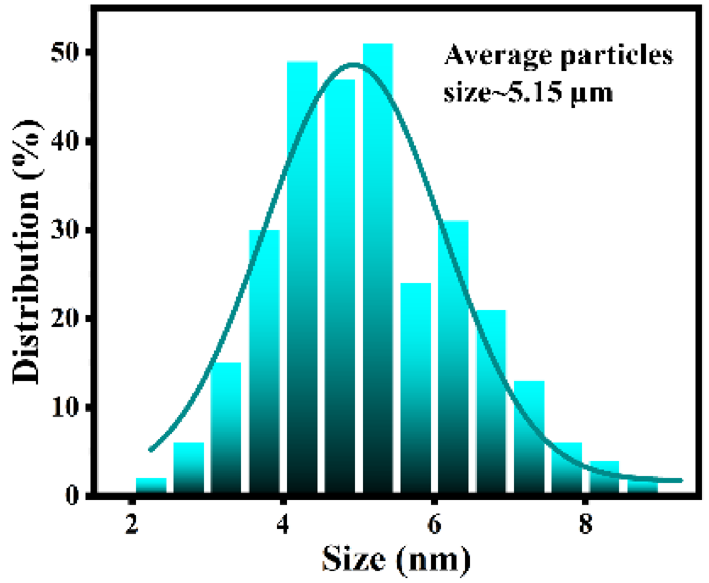

Figure S2 Particle size distribution of ECC powder.

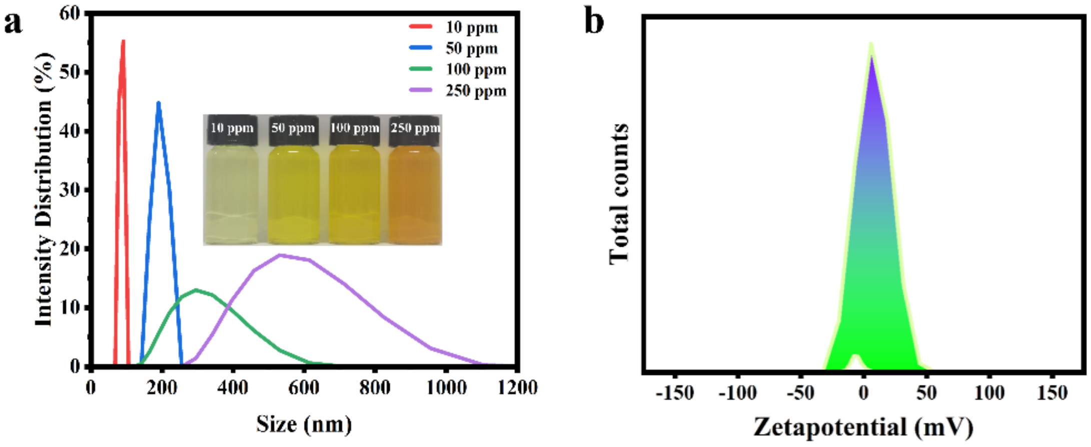

Figure S3 (a) Particle size distribution of ECC at different concentrations in ethanol. (b) Electrode potential diagram of ECC.

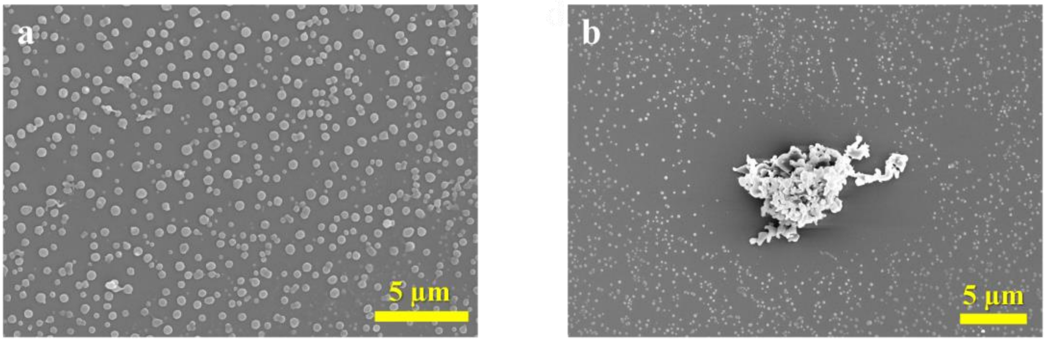

Figure S4 SEM image of (a) nanoscale spheres, (b) micrometer-scale structures in ECC dispersion with a concentration of 500 ppm.

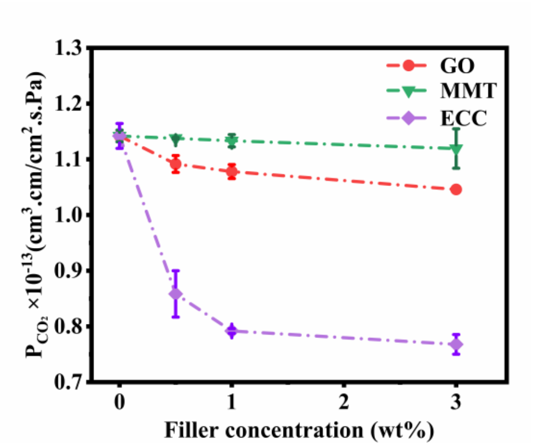

Figure S5 CO_2 permeability of composite films with different filling ratios. ECC/HDPE (prepared by melt-blending), GO/HDPE (prepared by solution-blending), MMT/HDPE (prepared by ball-milling). Measurement data are presented as mean ± SD with n = 3.

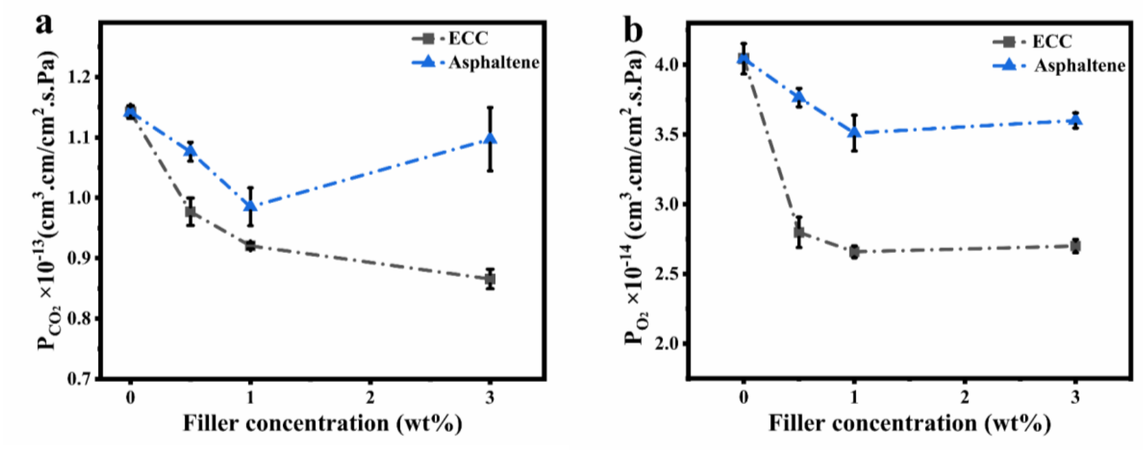

Figure S6 Gas permeability results of HDPE composite film prepared by solution method (a) CO_2. (b) O_2. Measurement data are presented as mean ± SD with n = 3.

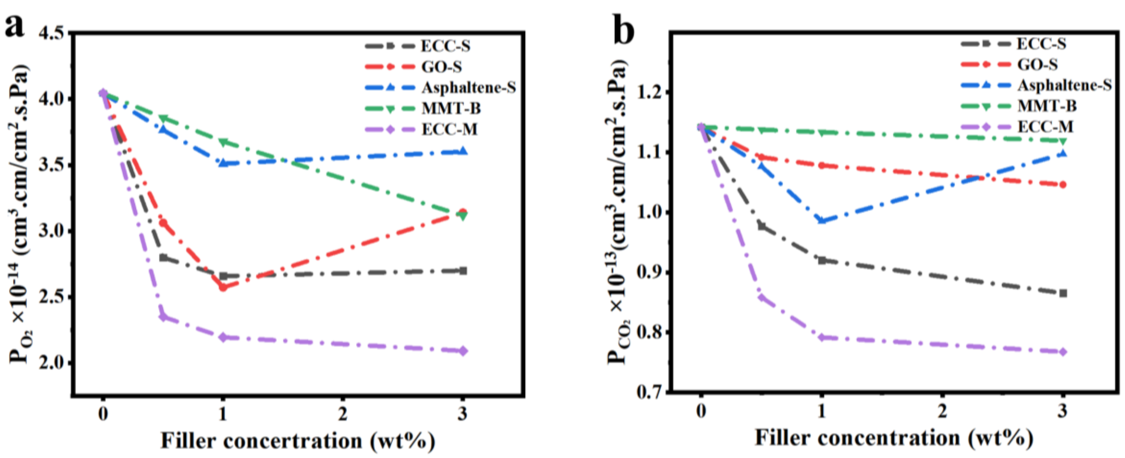

Figure S7 Gas permeability results of HDPE composite films prepared by melt-blending, solution-blending, and ball-milling methods (a) O_2. (b) CO_2. (M: melt-blending, S: solution-blending, B: ball-milling)

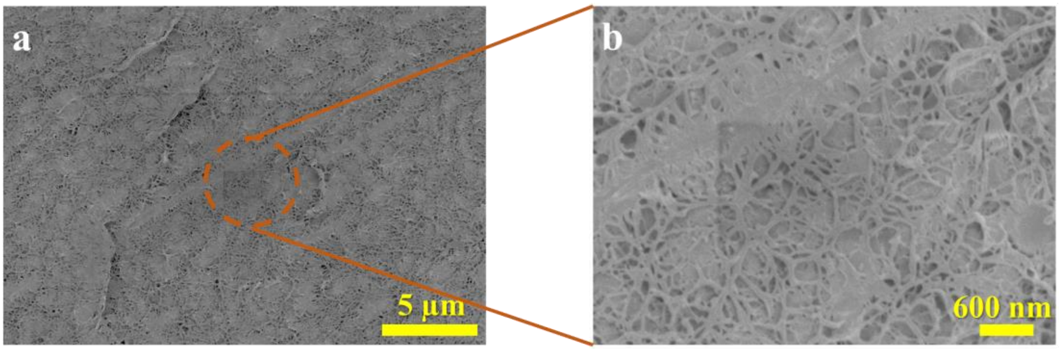

Figure S8 SEM images obtained at different magnifications of the cross-section of HDPE before aging. (a) ×5k. (b) ×22k. Continuous network structure can be observed in the unaged microstructure of the pure HDPE film.

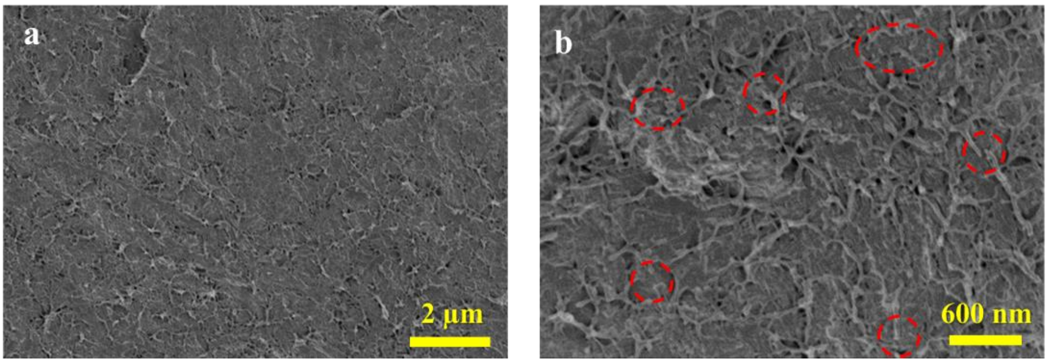

Figure S9 SEM images obtained at different magnifications of the cross-section of HDPE after aging for 60 hours. (a) ×5k. (b) ×22k. The molecular chain of HDPE is broken under ultraviolet irradiation, resulting in a discontinuous network structure of HDPE after aging (The broken molecular chains are shown in the red circles).

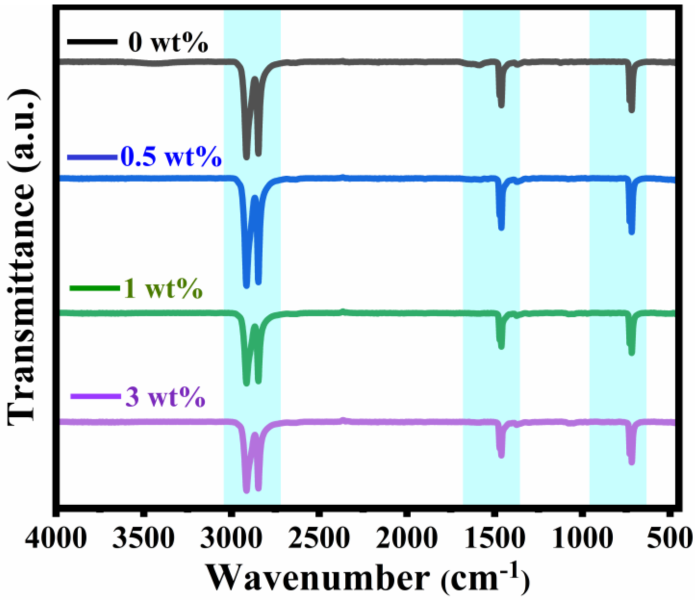

Figure S10 FT-IR spectra of ECC/HDPE with different ECC loadings.

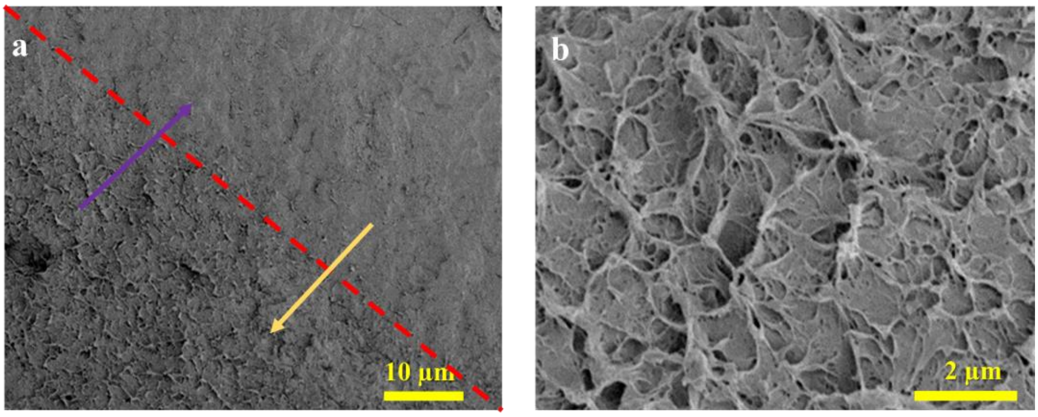

Figure S11 (a) SEM image of 1 wt% ECC/HDPE after aging for 60 hours (The red dashed line represents the boundary line, the purple arrow represents the direction of ultraviolet light irradiation, and the yellow arrow represents the direction of ECC movement). (b) SEM image of "ribbon-like structure" of HDPE molecular chain.
